# Frequency of Major Transmitted Integrase Resistance in Poland Remains Low Despite Change in Subtype Variability

**DOI:** 10.3390/v16101597

**Published:** 2024-10-11

**Authors:** Kaja Mielczak, Karol Serwin, Anna Urbańska, Bogusz Aksak-Wąs, Malwina Karasińska-Cieślak, Elżbieta Mularska, Adam Witor, Paweł Jakubowski, Maria Hlebowicz, Monika Bociąga-Jasik, Elżbieta Jabłonowska, Aleksandra Szymczak, Bartosz Szetela, Władysław Łojewski, Miłosz Parczewski

**Affiliations:** 1 Department of Infectious, Tropical Diseases and Immune Deficiency, Pomeranian Medical University in Szczecin, 71455 Szczecin, Poland; 2 Outpatient Clinic for AIDS Diagnostics and Therapy, Specialistic Hospital in Chorzow, 41500 Chorzow, Poland; 3 Infectious Diseases Gdansk, Pomeranian Hospitals, 80214 Gdansk, Poland; 4 Department of Infectious Diseases, Medical University of Gdansk, 81519 Gdansk, Poland; 5 Department of Infectious Diseases, Jagiellonian University Medical College, 30688 Krakow, Poland; 6 Department of Infectious Diseases and Hepatology, Medical University of Lodz, 91347 Lodz, Poland; 7 Department of Infectious Diseases, Liver Diseases and Acquired Immune Deficiencies, Wroclaw Medical University, 51149 Wroclaw, Poland; 8 Department of Infectious Diseases, University Hospital in Zielona Gora, 65046 Zielona Gora, Poland

**Keywords:** HIV, integrase strand transfer inhibitors, transmitted drug resistance, resistance mutations, clustering

## Abstract

With the widespread use of integrase inhibitors and the expanding use of long-acting cabotegravir in both pre-exposure prophylaxis and antiretroviral treatment, molecular surveillance on the transmission of integrase resistance has regained clinical significance. This study aimed to determine the frequency of INSTI-transmitted drug resistance mutations (DRMs) among treatment-naïve individuals in Poland from 2016 to 2023. INSTI resistance was analyzed in 882 antiretroviral treatment-naïve individuals using Sanger sequencing. Integrase DRMs were defined based on the Stanford HIV drug resistance database scores. Phylogeny was used to investigate subtyping and clustering. For the analysis of time-trends, logistic regression was used. Major (E138K and R263K) integrase mutations were detected in 0.45% of cases with minor resistance observed in 14.85%, most commonly (13.95%) E157Q. Overall, no major clusters of transmitted drug resistance were identified, and the transmission of E157Q showed a decreasing trend (*p* < 0.001). While the frequency of sub-subtype A6 increased, it was predominantly found among migrants and associated with L74 mutations. The frequency of major integrase-transmitted DRMs remains low, despite the changes in subtype variability. Surveillance of changing HIV molecular variation patterns is vital from the perspective of the optimal use of integrase inhibitors, especially due to expanding long-acting cabotegravir implementation.

## 1. Introduction

The introduction of integrase strand transfer inhibitors (INSTIs) has significantly improved the therapeutic options for treating HIV-1 infection, due to their high efficacy, decreased number of drug–drug interactions and faster inhibition of viral replication compared to other groups of antiretroviral agents [1,2,3,4]. First introduced integrase inhibitors (raltegravir and elvitegravir) were characterized by a lower genetic barrier to the selection of resistance [5,6,7]. Currently, most commonly used antiretroviral regiments include second generation integrase inhibitors, such as dolutegravir or bictegravir with a higher genetic barrier and infrequent selection of drug resistance in both in vitro studies and real-life cohorts [8,9,10,11]. Dolutegravir is also recommended as the global first line regimen by WHO recommendations [12]. Recently, with the introduction and expanding use of long-acting injectable cabotegravir in both pre-exposure HIV prophylaxis and antiretroviral treatment, which, despite its high efficacy, is associated with the selection of drug resistance in cases of virologic failure, the scientific interest in integrase resistance has been rekindled [13,14,15,16]. However, drug resistance testing for INSTIs is not routinely recommended or performed before the initiation of antiretroviral therapy.

Some studies have shown that transmitted INSTI drug resistance mutations (DRMs) are observed in 0.1–3% of naïve patients [17,18,19]. Transmission of major DRMs remains infrequent [20,21], while minor mutations and polymorphisms are observed more frequently but usually affect drug susceptibility or viral replicative capacity only when emerging in combination with major resistance variants [6,22]. Furthermore, the frequency of mutations may depend on the subtype or specific population [23,24]. A good example is the L74I polymorphism, which is characteristic of sub-subtype A6 and occurs in up to 90% of individuals [25]. In a study conducted by Hu et al. [26], it was demonstrated that L74I, in combination with mutations at positions 118, 140, 148 and 263, conferred a greater replication capacity to the sub-subtype A6.

In Poland, HIV prevalence used to be low, with the epidemic mainly driven by transmissions among men who have sex with men with a predominance of subtype B. However, since the outbreak of full-scale war in Ukraine in 2022, with the influx of war refugees, there has been a notable shift in the clinical and molecular patterns of the epidemic. This includes a rise in the number of heterosexually infected women under care and a significant increase in sub-subtype A6 transmissions and frequency [27]. Of note, the majority of refugees were already diagnosed in their home country and treated with a tenofovir disoproxil/emtricitabine/dolutegravir single tablet regimen; however, a notable proportion were newly diagnosed after arriving in Poland [28]. Studies on drug resistance transmission and selection in Ukraine are infrequent, while these new epidemiological trends may notably affect the patterns of drug resistance transmission in Poland. Also, there is a notable gap in the data on the recent transmission of integrase drug resistance, with the last available datasets encompassing 2010–2015 and a specific small group of blood donors from 2009 to 2017 [29,30]. Given the expanding use of integrase inhibitors in recent years and the above-mentioned differences in epidemiological patterns, in this study, we wished to determine the prevalence and time trends in transmitted DRMs associated with integrase inhibitors among treatment-naïve individuals living with HIV-1 in Poland.

## 2. Materials and Methods

### 2.1. Study Population

The study included 882 antiretroviral treatment-naïve people newly diagnosed with HIV-1 from 7 Polish centers (Szczecin, Chorzów, Gdańsk, Kraków, Łódź, Wrocław, Zielona Góra) from whom integrase sequences were obtained in the years of 2016–2023. Plasma samples were collected during the initiation of care (during hospitalization or their first visit to the HIV treatment center), before the introduction of antiretroviral treatment. The samples were subsequently sent to the Clinical Laboratory at the Department of Infectious, Tropical Diseases and Immune Deficiency, Pomeranian Medical University in Szczecin, where Sanger sequencing was performed. The number of sequences by year of diagnosis was as follows: 2016-88, 2017-111, 2018-293, 2019-132, 2020-124, 2021-44, 2022-38, 2023-52. The collected data included gender, age at diagnosis (first positive Western Blot test result), CD4 T-cell count and HIV-1 viral load at care entry, CDC clinical stage at care entry (AIDS vs. non-AIDS), and the route of transmission (as determined by the patient).

### 2.2. Sequencing and Subtyping

HIV RNA of samples collected from 2016 to 2022 were isolated using the Viroseq kit (Viroseq 2.8 and 2.9, Abbott Molecular, Abbott Park, IL, USA) according to the manufacturer’s protocol, while the QIAamp^®^ Viral RNA Mini Kit was used to isolate samples from 2023. The HIV-1 integrase region (866 b.p.) was amplified and sequenced with reagents and conditions according to the method outlined by Laethem et al. [31]. Amplicons obtained by the nested PCR method were used for sequencing using standard techniques with BigDye technology on an ABI 3500 platform (Applied Biosystems, Foster City, CA, USA). Sequence assembly was performed with the Recall online tool (https://recall.bccfe.ca, accessed on 16 August 2024).

The subtype was initially identified using the COMET tool (https://comet.lih.lu/, accessed on 20 August 2024) and the HIV BLAST algorithm (https://www.hiv.lanl.gov/content/sequence/BASIC_BLAST/basic_blast.html, accessed on 20 August 2024), and confirmed by phylogenetic analyses.

The accession numbers of the sequence data from this article have been deposited in GenBank and are available in the Supplementary Data.

### 2.3. Drug Resistance Interpretation

Drug resistance mutations were categorized according to the Stanford HIV drug resistance algorithm version 9.6 [32]. Each drug resistance mutation or combination has an assigned penalty score. We assumed that major DRMs have a score > 10, while minor mutations have a score of 10. Additionally, according to the Stanford interpretation, sequences with a resistance level ≥ 3 (indicating low, intermediate or high-level resistance) were considered resistant in our study. Due to the high number of sequences with sub-subtype A6, we have also included an analysis of the frequency of the L74I polymorphism, which is not associated with drug resistance according to the Stanford HIV database, but has been observed to influence the replicative capacity in cases with selected major integrase resistance mutations [26].

### 2.4. Statistical Analysis

Statistical comparisons were performed using the Chi^2^ test for nominal variables using Statistica 13.1 (StatSoft, Warsaw, Poland) software. Using R 4.4.0 statistical software, logistic regression was employed to analyze the distribution of DRMs and subtypes over time.

### 2.5. Phylogenetic Analyses

For our study, we analyzed the HIV-1 pol gene fragment corresponding to the integrase region, spanning nucleotide positions from 4230 to 5096 of the HXB2 reference genome. We focused on lineage B and sub-subtype A6 sequences, excluding other subtypes due to their limited sample sizes. Sequences containing more than 5% ambiguous bases were discarded from the alignment. The final dataset included 605 sequences of subtype B and 205 of sub-subtype A6. Sequences were aligned against the reference sequence K03455.1 (HXB2) using Clustal Omega software for each subtype [33]. Maximum likelihood (ML) trees were inferred using IQ-TREE, employing the generalized time-reversible evolutionary model [34]. Putative transmission clusters were identified using ClusterPicker v1.332 [35], with a genetic distance threshold of 0.015 and branch support ≥ 0.90 according to the Shimodaira–Hasegawa approximate likelihood ratio test (SH-aLRT). The final phylogenetic trees were prepared and annotated in iTOL [36].

## 3. Results

### 3.1. Baseline Characteristics of the Patients and HIV-1 Subtypes

Most of the individuals were male, 748 (84.81%), with 762 being of Polish origin (86.39%), and a median age of 35 (interquartile range [IQR], 16–75) years (Table 1). The dominant mode of transmission was MSM, accounting for 487 (55.22%) cases. The median CD4 T-cell count was 349 cells/μL (range, 1–2253 cells/μL), while the median viral load among cases was 4.80 log10 RNA copies/mL. HIV-1 RNA load ranged from 1.91 log10 RNA copies to 7.39 log10 RNA copies/mL. The proportion of late diagnosed cases was 411 (50.18%).

The most common HIV-1 variant was subtype B (616, 69.84%), followed by A6 (215, 24.38%), C and CRF02_AG (12, 1.36%, each), D (6, 0.68%), A1 (4, 0.45%), CRF01_AE, G and F (2, 0.23%, each), and other recombinant forms (11, 1.25%). Further details can be found in Table S1. Additionally, the distribution of subtypes by year of diagnosis is available in Table S2. Subtype B was more common in men compared to subtypes A6 and non-B non-A6 (*p* < 0.001), which were more common in women (Figure S1). There were no differences in median CD4 lymphocyte counts between subtypes. Median HIV-1 viral load was lower in subtype B compared to A6 (*p* = 0.001). Furthermore, the frequencies of late HIV diagnosis and advanced HIV disease (AIDS) were similar among all subtypes. In terms of transmission route, subtype B was more prevalent among MSM compared to A6 (*p* < 0.001) and non-B non-A6 subtypes (*p* = 0.016), in which heterosexual transmission was more frequent (B vs. A6, non-B non-A6 *p* < 0.001). Considering the country of origin, subtype B was significantly more frequent among Poles compared to migrants (77.95% vs. 18.33%, *p* < 0.001), in whom sub-subtype A6 was more frequently observed (75.00% vs. 16.40%, *p* < 0.001).

### 3.2. Frequency of INSTI DRMs and Drug Resistance

#### Transmitted Integrase Drug Resistance Mutations

A total of 135 (15.31%) sequences with DRMs were found, with 4 cases with major drug resistance mutations and 131 with minor mutations. Importantly, most of these sequences were sensitive to all INSTIs or exhibited potential low-level resistance only to first-generation integrase inhibitors, EVG and RAL. Considering the definition of resistance (Stanford resistance level ≥ 3), 878 sequences (99.55%) were susceptible to all INSTIs, while only 2 of 4 resistant sequences (0.23%) showed resistance to all drugs in this class. Two sequences had low-level resistance to EVG and RAL, while in two additional individuals, intermediate resistance to EVG, BIC, CAB, DTG and low resistance to RAL were detected. A detailed list of mutations is presented in the following sections.

- Major DRMs

Major DRMs were identified in four sequences (0.45%)—each had one major mutation—two (0.23%) E138K and two (0.23%) R263K. All individuals were male Poles with subtype B. Details on sequences with major mutations are provided in Table S3.

- Minor DRMs

A total of 132 minor DRMs were detected in 131 sequences (14.85%), with 2 mutations (E157Q and L74M) identified in one sequence with sub-subtype A6. The prevalence of the DRMs and their associated subtypes were as follows: E157Q (123, 13.95%)—121 (93.18%) with subtype B, 1 (0.81%) with A6, 1 (0.81%) with CRF03_AB, L74M (5, 0.57%, all males with sub-subtype A6), T97A (3, 0.34%, all males with subtype B), D232N (1, 0.11%, male with subtype B).

The E157Q mutation was notably more common among subtype B compared to sub-subtype A6 (*p* < 0.001) and non-B non-A6 subtypes (*p* < 0.001). It was also more frequent in women (44, 32.84%) compared to men (79, 10.56%, *p* < 0.001) and was associated with injection drug use (37, 68.52%). It was less frequent among MSM (10, 2.05%, *p* < 0.001) and the heterosexual population (46, 21.40%, *p* < 0.001).

The L74M mutation was observed more frequently in the A6 subtype compared to the other subtypes (*p* < 0.001), but no differences were noted for gender or transmission route. Details on sequences with minor mutations are provided in Table S4.

Additionally, we examined the prevalence of the L74I polymorphism which was found in 226 (25.62%) sequences that had the following subtypes: 1 (0.44%) A1, 189 (83.63%) A6, 27 (11.95%) B, 4 (1.77%) C, 4 (1.77%) CRF02_AG, and 1 (0.44%) D. The difference in the prevalence of L74I mutation between sub-subtype A6 and non-A6 was statistically significant (*p* < 0.001). It was also observed more frequently among women (*p* < 0.001) and heterosexual infections (HET vs. MSM *p* = 0.002).

Regarding the origin, minor DRMs were significantly more common among Poles (*p* = 0.001), with 125 mutations detected (117 E157Q, 4 L74M, 3 T97A, and 1 D232N), while among 6 migrants (4 with subtype B, 1 with A6 and 1 with CRF03_AB), only 7 substitutions were found (5 E157Q and 1 E157Q + L74M).

### 3.3. Distribution of Integrase DRMs and Subtypes over Time

**Figure 1:**
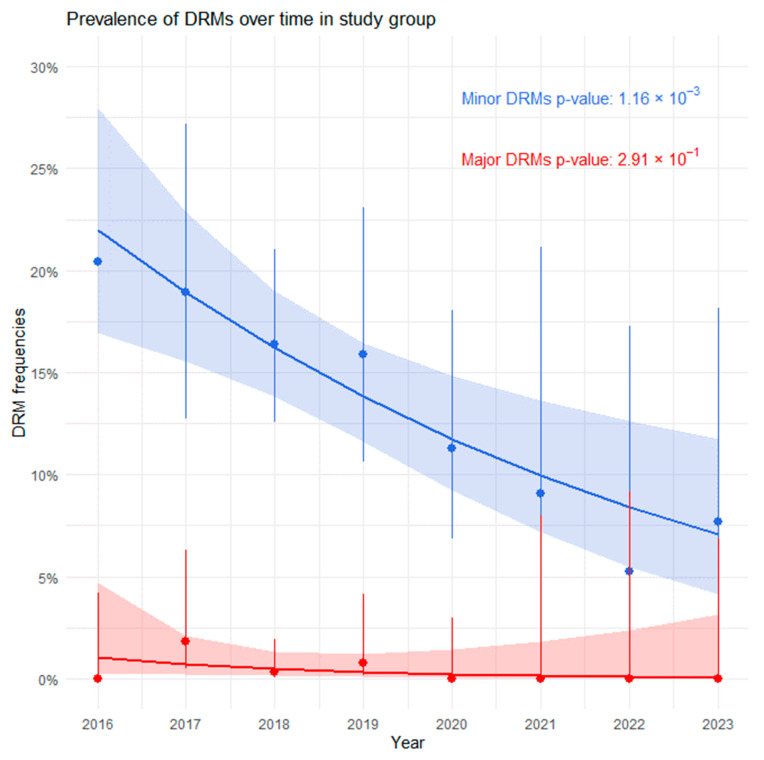
Logistic regression estimates for time trends between 2016 and 2023 are shown for major (red, dashed line) and minor (blue, solid line) DRMs. Dots represent the percentage per year, with vertical bars indicating 95% confidence intervals. The logistic regression trend lines are shown vertically, with shaded areas representing 95% confidence intervals for the regression estimate.

The distribution of mutations over time is shown in Figure 1. Due to the small number of major DRMs, there was no significant trend over time (OR 0.68, 95% CI 0.31–1.25, *p* = 0.291); however, for minor DRMs, a significant decrease was noted from 20.45% in 2016 to 7.69% in 2023 (OR 0.83, 95% Cl 0.74–0.92, *p* = 0.001).

**Figure 2:**
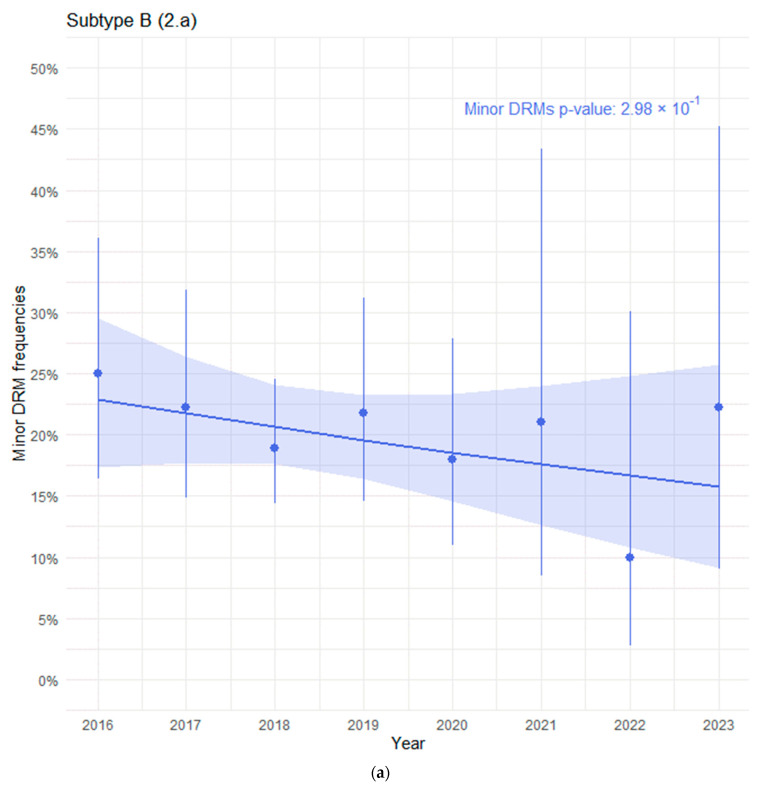

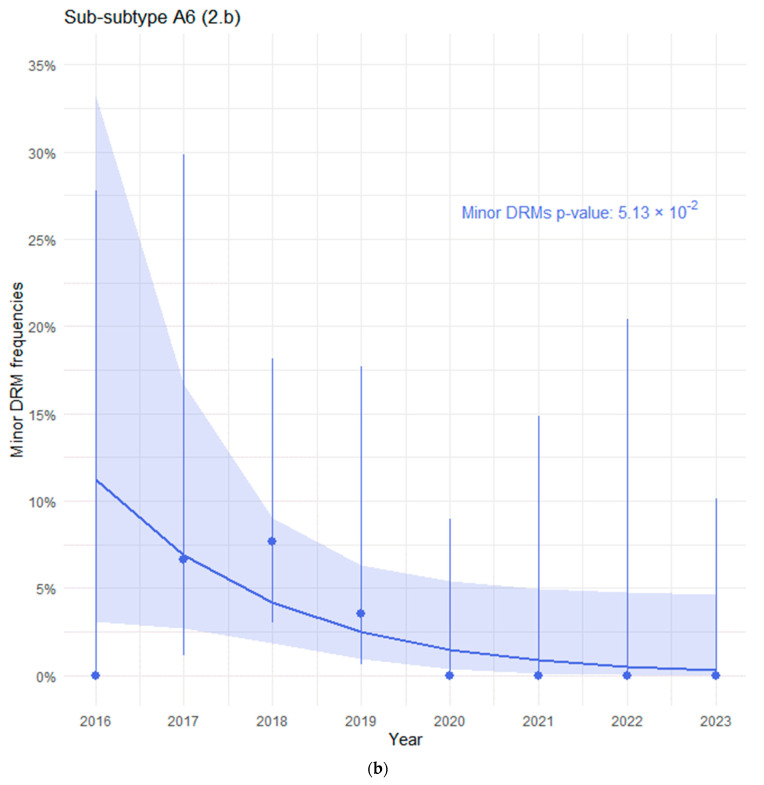
(**a**) Prevalence of minor DRMs over time among subtype B sequences. (**b**) Prevalence of minor DRMs over time among sub-subtype A6 sequences.

There was no significant trend in the prevalence of minor DRMs among cases with subtype B (Figure 2a, *p* = 0.298), but we observed a decreasing trend over time in the frequency of minor substitutions among sequences with sub-subtype A6 (Figure 2b, *p* = 0.051).

**Figure 3:**
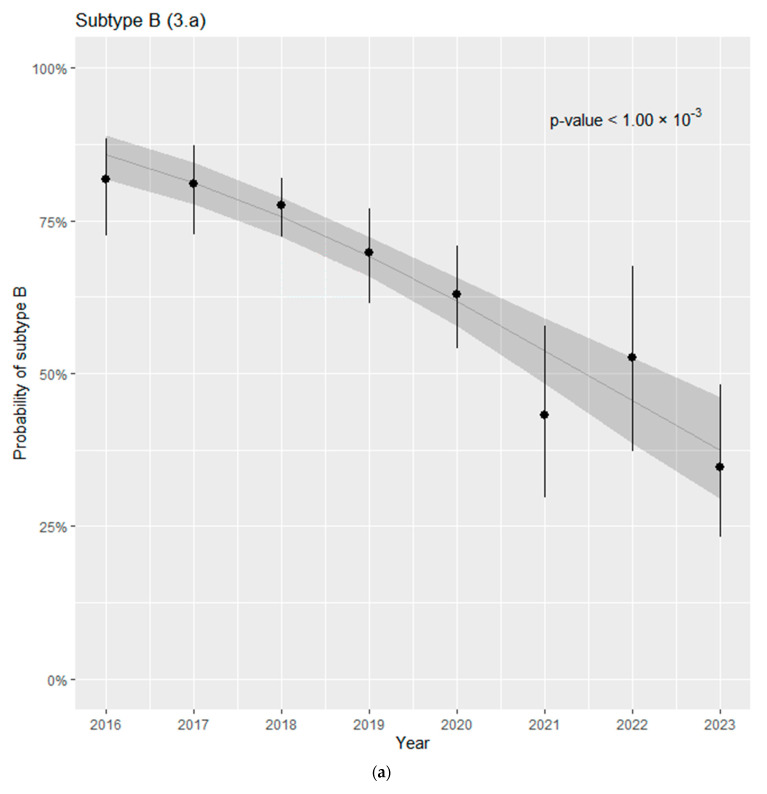

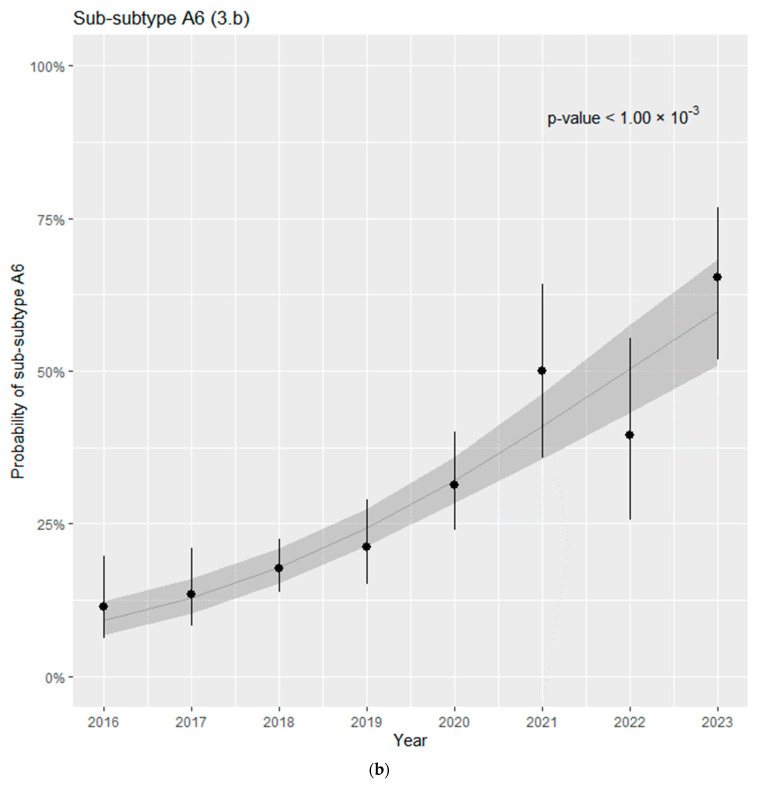
(**a**) Time trend in the prevalence of subtype B. (**b**) Time trend in the prevalence of sub-subtype A6.

Additionally, we estimated whether there were any temporal trends in the subtype prevalence. For subtype B (Figure 3a), we observed a statistically significant downward trend (*p* < 0.001), while for sub-subtype A6 (Figure 3b), there was a significant increase over time (*p* < 0.001).

Moreover, Figure S2 shows the downward temporal trends in the frequency of minor DRMs among men (a) (*p* = 0.030) and women (b) (*p* < 0.001). Furthermore, Figure S3 presents the temporal frequency of minor mutations by transmission route: MSM (a), HET (b), and IDU (c). For heterosexual cases, a statistically significant downward trend was observed (*p* < 0.001).

### 3.4. Sequence Clusters Including Clusterings of DRMs

**Figure 4:**
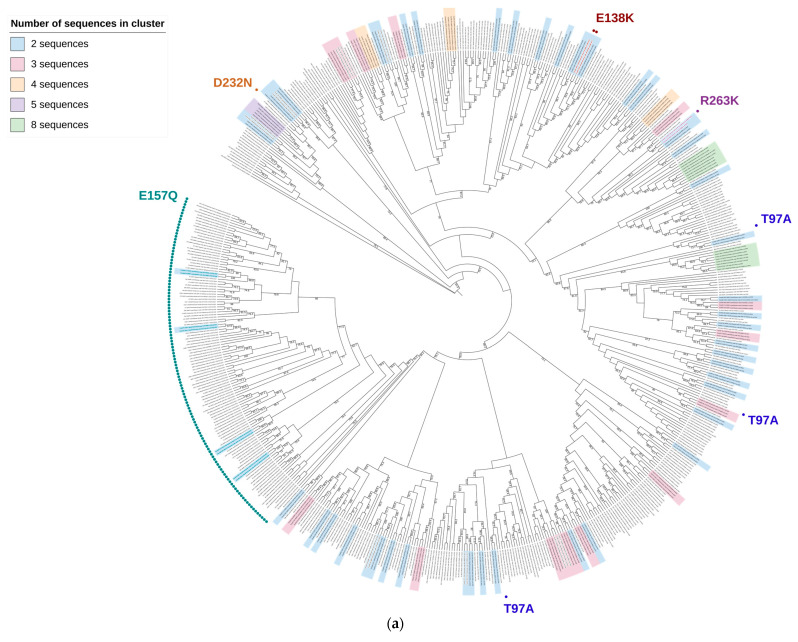

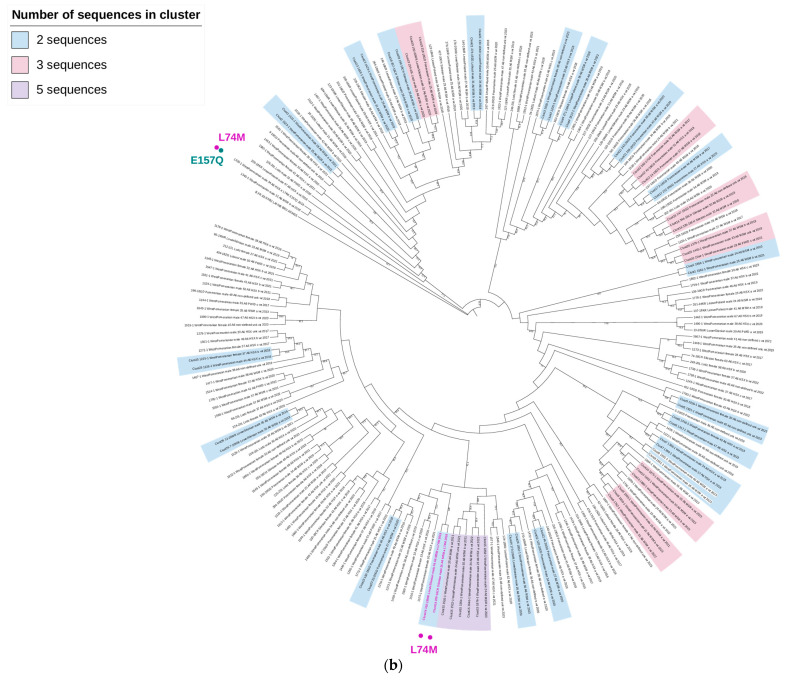
(**a**) Phylogenetic tree for subtype B. Sequences with DRMs are marked with dots. Clustering sequences are highlighted with different colors based on the number of sequences in the cluster, as indicated in the legend. Bootstrap values > 70% are shown at the branch nodes. (**b**) Phylogenetic tree for sub-subtype A6.

In total, 98 clusters were detected—72 for subtype B (Figure 4a) and 26 for sub-subtype A6 (Figure 4b).

For subtype B, there were 51 paired sequences, 6 of which had mutations: 1 with a R263K mutation, 1 with E138K and 4 with E157Q. Additionally, there were 15 clusters with 3 sequences, 3 clusters with 4 sequences, 1 cluster with 5 sequences and 2 clusters with 8 sequences.

For sub-subtype A6, there were 19 paired sequences, with 1 pair having an L74M polymorphic mutation. Moreover, six clusters with three sequences and one cluster with six sequences were found.

Overall, there were no major clusters of transmitted drug resistance.

## 4. Discussion

This study presents new data on HIV-1 patterns of integrase inhibitor resistance variants among treatment-naïve individuals from Poland, collected between 2016 and 2023. The last Polish publication on transmitted INSTI resistance included sequences from 2010 to 2015; therefore, this study is relevant due to the current, more frequent use of integrase inhibitors among Polish patients.

The overall frequency of transmitted drug resistance was 15.31%; however, major mutations constituted only 0.45%. These results are comparable to our earlier studies, in which major mutations were observed infrequently (in the 2012 study, one R263K mutations was detected, which was then classified as a minor mutation) [29,37]. Studies from other countries show that major DRMs are found in about 0.3–1.5% of newly diagnosed people living with HIV [19,38,39,40]. The two major mutations detected in our study were E138K and R263K. Substitution of E138K is detected in approximately 0.15% of individuals [38,41], and is selected in patients receiving RAL, EVG, CAB, and DTG. It confers resistance to INSTIs in combination with other mutations, although in our study it was detected alone and occurred in 0.23% of individuals. R263K, a nonpolymorphic mutation, occurs in ~0.10% of naïve individuals [38,41], it is selected in vitro by EVG, DTG, BIC, and CAB and reduces sensitivity to DTG, BIC and CAB by about 2-fold [11,42]. In our study, similarly to E138K, it was identified in 0.23% of sequences. Both E138K and R263K mutations can occur in conjunction with APOBEC G-to-A hypermutation; however, it was not detected in the sequences containing these mutations. Since 2020, we have not found any major DRMs, and in previous years, there was no significant trend due to the low number of mutations.

Minor mutations were detected in 14.85% of sequences, with the vast majority being the E157Q polymorphism (13.95%). Other detected DRMs included L74M (0.57%), T97A (0.34%), and D232N (0.11%). L74M is a polymorphic mutation that occurs in 1–5% of individuals depending on the subtype, and is more frequently observed in non-B subtypes [43,44]. It reduces sensitivity to INSTIs only in combination with other mutations [11,45], but in our study, it occurred alone and was observed only in sub-subtype A6.

Various studies show that the E157Q polymorphism is detected in about 2.5% of treatment-naïve patients [19,38,46]. However, depending on the subtype, its frequency may vary from 1.7% to 5.6% [47]. In the Polish population, this mutation is much more frequent—in 2012, it was observed in 28.8% of the population [37], and in 2017 in 19.19% [29]. The association between the E157Q mutation and reduced sensitivity to INSTIs is weak and this variant alone does not affect sensitivity to INSTIs in vitro [48]. In our study, only one combination with the E157Q mutation, E157Q + L74M, was observed, but it had no significant effect on resistance. However, according to the Stanford database, it may show potentially low resistance to raltegravir and elvitegravir, while the French ANRS database associates this substitution with full resistance to first-generation INSTIs [49]. Studies in real-world settings show that a small percentage of patients who carry the E157Q variant experience virological failure when treated with RAL or EVG [46,50].

Additionally, in our study, E157Q was more frequently observed among subtype B, women and injection drug users, which is in line with a previous Polish study [29]. The significant prevalence of the E157Q mutation in the subtype B population in Poland might indicate a weak founder effect, potentially resulting from a virus that was previously transmitted extensively among IDUs. The higher prevalence of this substitution in IDUs may be also related to their weaker adherence and more frequent virological failure, predisposing them to the development of drug resistance [51].

In Poland, a continuous decrease in the frequency of minor mutations (mainly E157Q) has been observed—in 2012, it was 38.5% [37], dropped to 21.5% in 2017 [29], and currently it is 14.85%. We did not observe significant differences in the distribution of minor mutations by year for gender or subtype. Regarding the route of transmission, a statistically significant downward trend is observed in heterosexual infections, which may be related to the fact that the E157Q variant mainly occurs in subtype B associated with MSM infections. Since we observed a systematic decrease in the prevalence of subtype B in Poland, the frequency of the E157Q mutation, which is a polymorphic variant for this subtype, is also decreasing. For sub-subtype A6, of which prevalence is significantly increasing, the E157Q mutation is not polymorphic and was detected in only one individual (0.81%).

Furthermore, the E157Q polymorphism clustered within subtype B—four pairs of sequences with this mutation were detected. Other studies have also observed clustering of this mutation [37,52]. In a previous Polish study, the E157Q variant was present in a large cluster of 33 sequences, which were transmitted to heterosexually infected female partners and associated with a history of injection drug use [29]. We did not observe such a trend in our clusters, as transmission route was unknown for four individuals, while the remaining consisted of two MSM, one IDU, and one heterosexual transmission.

The change in the distribution of subtypes over the last few years is noteworthy. There is a clear downward trend in the prevalence of subtype B and an upward trend in sub-subtype A6. In our previous study conducted by Serwin et al. [53], it was estimated that from 2015 to 2019, sub-subtype A6 was present in 8.66% of cases, while in the current study, it has already increased to 24.38%. This change is largely attributable to the Russian invasion of Ukraine, which in 2022, led to significant migration, primarily to Poland. Among the Polish population, sub-subtype A6 was found in 16.4% of cases, while in migrants it was present in 75.0% of cases. Although no major mutations were observed among migrants and the E157Q variant was rare, it is important to note that sub-subtype A6 is associated with mutations at position L74, which are linked to the development of resistance to cabotegravir. In our study, the L74I polymorphism was frequent.

The study has several limitations. There was no systematic sampling of patients for integrase resistance analyses as there is no national systematic drug resistance surveillance program. Polish centers provided materials collected from patients for primary transmitted drug-resistance analysis based on physicians’ request. Additionally, the study covers centers following up patients from approximately half the territory of Poland. Therefore, the study does not fully reflect the prevalence of integrase DRMs in the overall population, but remains the largest Polish cohort to date. It was also impossible to confirm whether all cases were truly treatment-naïve as it was self-reported by patients.

## 5. Conclusions

In conclusion, transmitted resistance to integrase inhibitors remains low, with a high frequency of the polymorphic E157Q mutation, which is more prevalent in the Polish population compared to other countries and primarily observed among females and injection drug users. The frequency of minor mutations continues to decrease, and their clustering is less frequent compared to previous years. It is important to highlight the significant shift in the subtype distribution in Poland. Sub-subtype A6 is becoming more common, and since it is primarily associated with mutations at position L74, it may affect resistance to long-acting cabotegravir. However, despite the changes in subtype variability, major integrase transmitted DRMs continue to be rare.

## Figures and Tables

**Table 1:** Characteristics of the study population.

	Total	Subtype B n = 616	Sub-Subtype A6 n = 215	Subtype Non-B, Non-A6 n = 51	*p* Value: B vs. A6	*p* Value: B vs. Non-A6, Non-B	*p* Value: A6 vs. Non-B, Non-A6
Gender, n (%)							
Male	748 (84.81)	552 (89.61)	159 (73.95)	37 (72.55)	<0.001	<0.001	0.839
Female	134 (15.19)	64 (10.39)	56 (26.05)	14 (27.45)			
Age (years), median (IQR)	35 (16–75)	35 (16–75)	35 (21–73)	38 (18–68)			
CD4 T-cell count (cells/μL), median (IQR) ^a^	349 (1–2253)	355 (1–2253)	335 (1–1296)	246 (1–1579)			
<200	245 (30.55)	165 (29.52)	60 (30.77)	20 (41.67)	0.742	0.079	0.150
≥200	557 (69.45)	394 (70.58)	135 (69.23)	28 (58.33)			
HIV-1 viral load (log10 RNA copies/mL), median (IQR) ^b^	4.80 (1.91–7.39)	4.74 (2.22–7.24)	5.05 (1.91–7.00)	4.91 (2.55–7.39)			
<5	467 (58.01)	344 (61.87)	100 (48.78)	23 (52.27)	0.001	0.209	0.674
≥5	338 (41.99)	212 (38.13)	105 (51.22)	21 (47.73)			
Late diagnosis (CD4 T-cell count (cells/μL) <350 or AIDS ^c^	411 (50.18)	279 (49.12)	106 (52.48)	26 (53.06)	0.413	0.596	0.941
Clinical category at HIV diagnosis (AIDS vs. non-AIDS) ^d^							
AIDS	150 (19.13)	106 (19.38)	35 (18.42)	9 (19.15)	0.772	0.969	0.908
Non-AIDS	634 (80.87)	441 (80.62)	155 (81.58)	38 (80.85)			
Risk exposure, n (%) ^e^							
MSM *	487 (55.22)	364 (69.07)	101 (54.01)	22 (51.16)	<0.001	0.016	0.736
HET **	215 (24.38)	117 (22.20)	78 (41.71)	20 (46.51)	<0.001	<0.001	0.566
IDU ***	54 (6.12)	45 (8.54)	8 (4.28)	1 (2.33)	0.056	0.150	0.551
MTC ****	1 (0.11)	1 (0.19)	0	0	0.797		

Data available for: ^a^ 802 individuals, ^b^ 805 individuals, ^c^ 819 individuals, ^d^ 784 individuals, ^e^ 757 individuals. * Men who have Sex with Men, ** Heterosexual, *** Injection drug user, **** Mother-to-child.

## Data Availability

The accession numbers of the sequence data from this article have been deposited in GenBank and are available in the Supplementary Data. The data presented in this study are available from the corresponding author upon reasonable request.

## Supplementary Materials

The following supporting information can be downloaded at: https://www.mdpi.com/article/10.3390/v16101597/s1: List of GenBank accession numbers of the sequence data; Table S1: Distribution of subtypes among study group; Table S2: Subtype distribution by year of diagnosis; Table S3: Detailed information on sequences with major mutations; Table S4: Detailed information on sequences with minor mutations; Figure S1: Subtype distribution among men and women; Figure S2: Prevalence of minor DRMs over time among men (a) and women (b); Figure S3: Prevalence of minor DRMs over time among MSM (a), HET (b) and IDU (c).
