# Supplementary material for: Frequency of Major Transmitted Integrase Resistance in Poland Remains Low Despite Change in Subtype Variability
Source: Viruses. 2024 Oct 11;16(10):1597. doi: 10.3390/v16101597 (PMC11512334; doi:10.3390/v16101597)
Supplement: Supplementary file 1 [file viruses-16-01597-s001.zip › viruses-3232212-supplementary.pdf]

The GenBank accession numbers of the sequence data from this article are as follows:

OP301658, OP301660, OP301669, OP301673, OP301683, OP301685, OP301694, OP301696, OP301707-OP301708, OP301718, OP301725, OP301735, OP301743, OP301745-OP301746, OP301755, OP301757-OP301758, OP301766, OP301781, OP301783, OP301793, OP301801-OP301802, OP301811, OP301825, OP301827-OP301840, OP301842-OP301850, OP301852-OP301876, OP301878-OP301879, OP301881-OP301882, OP301884-OP301885, OP301887-OP301888, OP301890-OP301926, OP301928-OP301929, OP301931-OP301942, OP301944-OP301965, OP301967-OP301996, OP301998-OP302014, OP302016-OP302017, OP302019-OP302021, OP302023-OP302039, OP302041-OP302047, OP302049-OP302059, OP302061-OP302065, OP302067-OP302068, OP302070-OP302071, OP302073-OP302080, OP302083-OP302085, OP302087-OP302090, OP302092-OP302095, OP302097, OP302099-OP302104, OP302106-OP302108, OP302110-OP302112, OP302115-OP302118, OP302120-OP302121, OP302123, OP302126-OP302128, OP302131, OP302133-OP302138, OP302140-OP302145, OP302147-OP302154, OP302156-OP302167, OP302170-OP302174, OP302176, OP302178-OP302183, OP302185-OP302187, OP302190, OP302192-OP302199, OP302201, OP302203-OP302205, OP302208-OP302209, OP302211-OP302212, OP302215-OP302219, OP302221-OP302230, OP302232, OP302235-OP302256, OP302258, OP302263-OP302269, OP302272-OP302273, OP302275-OP302278, OP302281-OP302285, OP302287-OP302294, OP302296-OP302304, OP302306, OP302308-OP302310, OP302312-OP302318, OP302320-OP302326, OP302328-OP302334, OP302336-OP302341, OP302343-OP302353, OP302355-OP302360, OP302363-OP302367, OP302369-OP302376, OP302378-OP302382, OP302384-OP302385, OP302387, OP302389, OP302391-OP302395, OP302397-OP302402, OP302404, OP302408-OP302409, OP302411-OP302415, OP302417-OP302418, OP302420-OP302429, OP302431-OP302434, OP302436-OP302437, OP302439-OP302442, OP302444, OP302446-OP302454, OP302456, OP302458-OP302459, OP302462-OP302464, OP302468-OP302470, OP302472-OP302481, OP302483-OP302488, OP302490-OP302492, OP302494, OP302496-OP302498, OP302500-OP302505, OP302507-OP302509, OP302513-OP302519, OP302521-OP302528, OP302530, OP302536-OP302538, OP302540-OP302541, OP302543, OP302546, OP302548, OP302550-OP302551, OP302553-OP302554, OP302556-OP302558, OP302563, OP302566-OP302567, OP302573-OP302576, OP302581-OP302583, OP302586, OP302591, OP302594, OP302596, OP302599-OP302600, OP302605, OP302610, OP302612, OP302616, OP302618-OP302621, OP302623, OP302625, OP302628, OP302630, OP302638-OP302639, OP302642-OP302643, OP302648, OP302651, OP302658, OP302662, OP302670, OP302676, OP302680, OP302683-OP302684, OP302689, OP302696, OP302700, OP302703-OP302705, OP302708, OP302711, OP302713, OP302716, OP302720-OP302722, OP302727.

Table S1. Distribution of subtypes among study group

Subtype	n (%)
B	616 (69.84)
A6	215 (24.38)
A1	4 (0.45)
C	12 (1.36)
D	6 (0.68)
F	2 (0.23)
G	2 (0.23)
**CRFs**	
CRF01_AE	2 (0.23)
CRF02_AG	12 (1.36)
CRF03_AB	1 (0.11)
CRF06_CPX	1 (0.11)
CRF07_BC	1 (0.11)
CRF09_CPX	1 (0.11)
CRF12_BF	2 (0.23)
CRF19_CPX	1 (0.11)
CRF47_BF	1 (0.11)
CRF63_02A1	1 (0.11)
**URFs**	
A1B	1 (0.11)
A6B	1 (0.11)

CRFs- circulating recombinant forms

URFs- unique recombinant forms

Table S2. Subtype distribution by year of diagnosis

Subtype	Year of diagnosis							
	2016	2017	2018	2019	2020	2021	2022	2023
**A6**	10 (11.36%)	15 (13.51%)	52 (17.75%)	28 (21.21%)	39 (31.45%)	22 (50.00%)	15 (39.47%)	34 (65.38%)
**A1**	1 (1.14%)	0	2 (0.68%)	1 (0.76%)	0	0	0	0
**B**	72 (81.82%)	90 (81.08%)	227 (77.47%)	92 (69.70%)	78 (62.90%)	19 (43.18%)	20 (52.63%)	18 (34.62%)
**C**	0	0	5 (1.71%)	3 (2.27%)	2 (1.61%)	1 (2.27%)	1 (2.63%)	0
**D**	0	1 (0.90%)	1 (0.34%)	1 (0.76%)	1 (0.81%)	1 (2.27%)	1 (2.63%)	0
**F**	0	1 (0.90%)	0	0	1 (0.81%)	0	0	0
**G**	0	0	0	1 (0.76%)	0	1 (2.27%)	0	0
**CRF01_AE**	0	0	0	1 (0.76%)	1 (0.81%)	0	0	0
**CRF02_AG**	3 (3.41%)	2 (1.80%)	4 (1.37%)	2 (1.52%)	1 (0.81%)	0	0	0
**Other RFs**	2 (2.27%)	2 (1.80%)	2 (0.68%)	3 (2.27%)	1 (0.81%)	0	1 (2.63%)	0

Figure S1. Subtype distribution among men and women

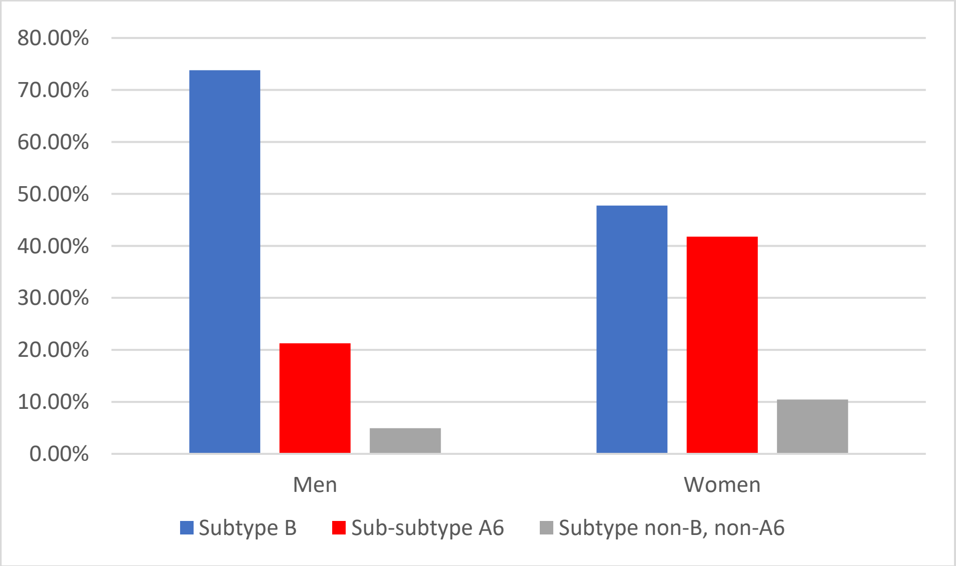

Table S3. Detailed information on sequences with major mutations

Level of resistance					INSTI major mutations	INSTI accessory mutations	Subtype	Sex	Transmission route	CDC	Age	CD4 count (cells/μL)	HIV viral load (log10 RNA copies/mL)
BIC	CAB	DTG	EVG	RAL									
P	P	P	L	L	E138K	None	B	M	MSM	A	46	469	4.30
I	I	I	I	L	R263K	None	B	M	HET	C	47	10	4.14
P	P	P	L	L	E138K	None	B	M	MSM	A	57	430	3.76
I	I	I	I	L	R263K	None	B	M	UNK	UNK	41	UNK	UNK

P- Potential low-level resistance
L- Low-level resistance
I- Intermediate-level resistance

Table S4. Detailed information on sequences with minor mutations

Level of resistance					INSTI major mutations	INSTI accessory mutations	Subtype	Sex	Transmission route	CDC	Age	CD4 count (cells/μL)	HIV viral load (log10 RNA copies/mL)
BIC	CAB	DTG	EVG	RAL									
S	S	S	S	S	None	L74M	A6	M	MSM	A	34	379	6.08
S	S	S	P	P	None	E157Q	B	F	HET	C	32	78	5.67
S	S	S	P	P	None	E157Q	B	M	IDU	B	57	238	5.53
S	S	S	P	P	None	E157Q	B	F	HET	A	38	105	4.42
S	S	S	P	P	None	E157Q	B	M	IDU	A	53	295	4.00
S	S	S	P	P	None	E157Q	B	M	IDU	C	42	137	4.90
S	S	S	P	P	None	E157Q	B	M	UNK	UNK	32	294	5.38
S	S	S	P	P	None	E157Q	B	F	HET	B	26	328	5.63
S	S	S	P	P	None	E157Q	B	M	HET	C	37	198	5.70
S	S	S	P	P	None	E157Q	B	F	HET	B	32	103	4.61
S	S	S	P	P	None	E157Q	B	F	IDU	C	35	8	5.46
S	S	S	P	P	None	E157Q	B	F	HET	A	28	350	4.33
S	S	S	P	P	None	E157Q	B	M	IDU	C	52	14	4.83
S	S	S	P	P	None	E157Q	B	F	UNK	A	40	95	5.41
S	S	S	P	P	None	E157Q	B	M	UNK	B	40	77	5.39
S	S	S	P	P	None	E157Q	B	M	IDU	C	33	98	5.61
S	S	S	P	P	None	E157Q	B	F	HET	A	41	250	4.76
S	S	S	P	P	None	E157Q	B	F	IDU	C	35	217	5.01
S	S	S	P	P	None	E157Q	B	F	HET	A	33	402	3.81
S	S	S	P	P	None	E157Q	B	M	HET	C	50	108	4.31
S	S	S	P	P	None	E157Q	B	F	HET	C	50	53	5.24
S	S	S	P	P	None	T97A	B	M	HET	A	61	370	4.07
S	S	S	P	P	None	E157Q	B	M	HET	A	43	60	5.00
S	S	S	P	P	None	E157Q	B	F	IDU	UNK	37	UNK	UNK
S	S	S	P	P	None	E157Q	B	M	UNK	UNK	58	229	2.36
S	S	S	P	P	None	E157Q	B	M	MSM	C	32	801	UNK
S	S	S	P	P	None	E157Q	B	F	IDU	B	39	213	3.93
S	S	S	P	P	None	T97A	B	M	MSM	A	28	715	5.06

S	S	S	P	P	None	E157Q	B	M	UNK	UNK	38	993	UNK
S	S	S	P	P	None	E157Q	B	M	IDU	B	38	557	UNK
S	S	S	P	P	None	E157Q	B	F	HET	B	35	464	6.00
S	S	S	P	P	None	E157Q	B	M	UNK	UNK	42	424	UNK
S	S	S	P	P	None	E157Q	B	M	IDU	A	24	348	6.49
S	S	S	P	P	None	E157Q	B	M	UNK	B	29	4	5.71
S	S	S	P	P	None	E157Q	B	F	UNK	UNK	23	UNK	UNK
S	S	S	P	P	None	L74M,E157Q	A6	M	HET	A	34	UNK	UNK
S	S	S	P	P	None	E157Q	B	M	UNK	UNK	64	58	UNK
S	S	S	P	P	None	E157Q	B	M	UNK	B	47	54	5.24
S	S	S	P	P	None	E157Q	B	F	IDU	B	30	653	4.53
S	S	S	P	P	None	E157Q	B	F	HET	A	28	690	4.19
S	S	S	P	P	None	E157Q	B	M	UNK	UNK	48	82	6.34
S	S	S	P	P	None	E157Q	B	M	IDU	A	38	505	4.49
S	S	S	P	P	None	E157Q	B	F	HET	A	30	212	4.36
S	S	S	P	P	None	E157Q	B	M	HET	A	50	137	4.80
S	S	S	P	P	None	E157Q	B	M	IDU	C	48	31	4.64
S	S	S	P	P	None	E157Q	B	F	HET	B	41	UNK	4.64
S	S	S	P	P	None	E157Q	B	M	UNK	C	48	50	5.72
S	S	S	P	P	None	E157Q	B	F	HET	C	35	28	5.78
S	S	S	P	P	None	E157Q	B	M	HET	A	44	4	5.67
S	S	S	P	P	None	E157Q	B	M	IDU	B	39	555	4.39
S	S	S	P	P	None	E157Q	B	M	IDU	C	25	118	5.99
S	S	S	P	P	None	E157Q	B	F	UNK	UNK	38	24	5.34
S	S	S	P	P	None	D232N	B	M	MSM	A	46	256	5.18
S	S	S	P	P	None	E157Q	B	F	HET	A	39	779	4.94
S	S	S	P	P	None	E157Q	B	F	IDU	A	35	UNK	UNK
S	S	S	P	P	None	E157Q	B	M	HET	C	52	44	5.39
S	S	S	P	P	None	E157Q	B	M	HET	A	31	355	4.47
S	S	S	P	P	None	E157Q	B	F	HET	A	23	296	5.28
S	S	S	P	P	None	E157Q	B	M	UNK	UNK	35	UNK	UNK
S	S	S	P	P	None	E157Q	B	M	HET	B	49	115	5.00
S	S	S	P	P	None	E157Q	B	M	MSM	B	29	136	5.69

S	S	S	P	P	None	E157Q	B	F	UNK	C	52	3	5.31
S	S	S	P	P	None	E157Q	B	M	IDU	C	42	504	4.04
S	S	S	P	P	None	E157Q	B	F	UNK	UNK	46	54	6.14
S	S	S	P	P	None	E157Q	B	M	IDU	A	50	377	3.98
S	S	S	P	P	None	E157Q	B	M	IDU	UNK	46	UNK	UNK
S	S	S	S	S	None	L74M	A6	M	MSM	A	37	610	4.89
S	S	S	S	S	None	L74M	A6	M	MSM	A	35	713	5.28
S	S	S	P	P	None	E157Q	B	M	HET	A	38	431	5.30
S	S	S	P	P	None	E157Q	B	M	MSM	A	45	448	4.37
S	S	S	P	P	None	E157Q	B	F	IDU	A	28	929	3.63
S	S	S	P	P	None	E157Q	B	M	HET	B	36	363	5.86
S	S	S	P	P	None	E157Q	B	F	UNK	C	35	11	UNK
S	S	S	P	P	None	E157Q	B	F	HET	B	36	142	3.82
S	S	S	P	P	None	E157Q	B	F	HET	B	40	286	3.94
S	S	S	P	P	None	E157Q	CRF03_AB	M	HET	A	56	627	4.54
S	S	S	P	P	None	E157Q	B	M	HET	A	36	155	4.70
S	S	S	P	P	None	E157Q	B	F	HET	B	40	58	UNK
S	S	S	P	P	None	E157Q	B	M	HET	A	55	508	3.44
S	S	S	P	P	None	E157Q	B	F	HET	A	23	449	3.88
S	S	S	P	P	None	E157Q	B	M	UNK	UNK	45	UNK	UNK
S	S	S	P	P	None	E157Q	B	M	HET	A	41	316	3.88
S	S	S	P	P	None	E157Q	B	M	IDU	B	44	10	5.68
S	S	S	P	P	None	E157Q	B	M	UNK	C	48	8	4.32
S	S	S	P	P	None	E157Q	B	M	MSM	A	35	524	3.30
S	S	S	P	P	None	E157Q	B	F	UNK	UNK	27	UNK	UNK
S	S	S	P	P	None	E157Q	B	M	IDU	B	36	UNK	UNK
S	S	S	P	P	None	E157Q	B	F	IDU	C	37	537	4.72
S	S	S	P	P	None	E157Q	B	M	IDU	A	44	637	4.26
S	S	S	P	P	None	E157Q	B	M	HET	C	45	98	UNK
S	S	S	P	P	None	E157Q	B	F	HET	B	41	76	5.06
S	S	S	P	P	None	E157Q	B	M	IDU	C	56	200	7.19
S	S	S	P	P	None	E157Q	B	M	MSM	A	29	1204	3.95
S	S	S	P	P	None	E157Q	B	F	UNK	C	38	14	4.62

S	S	S	P	P	None	E157Q	B	M	UNK	UNK	40	UNK	UNK
S	S	S	P	P	None	E157Q	B	F	IDU	UNK	47	UNK	UNK
S	S	S	P	P	None	E157Q	B	M	IDU	A	38	873	4.68
S	S	S	P	P	None	E157Q	B	M	UNK	UNK	41	UNK	UNK
S	S	S	P	P	None	E157Q	B	M	IDU	A	39	107	UNK
S	S	S	S	S	None	L74M	A6	M	MSM	A	37	280	5.09
S	S	S	P	P	None	E157Q	B	F	HET	A	41	205	4.82
S	S	S	P	P	None	E157Q	B	M	MSM	A	37	429	5.09
S	S	S	P	P	None	E157Q	B	M	HET	A	38	612	6.27
S	S	S	P	P	None	E157Q	B	M	MSM	A	20	421	5.81
S	S	S	P	P	None	E157Q	B	M	HET	A	19	522	3.84
S	S	S	P	P	None	E157Q	B	M	IDU	C	32	90	4.72
S	S	S	P	P	None	E157Q	B	M	IDU	B	42	724	3.87
S	S	S	P	P	None	E157Q	B	M	HET	A	27	128	4.03
S	S	S	P	P	None	E157Q	B	F	HET	C	46	48	5.05
S	S	S	P	P	None	E157Q	B	M	HET	C	56	146	5.74
S	S	S	P	P	None	E157Q	B	M	IDU	C	42	95	5.24
S	S	S	P	P	None	E157Q	B	F	MTC	UNK	18	UNK	UNK
S	S	S	P	P	None	E157Q	B	M	IDU	C	44	68	3.18
S	S	S	P	P	None	E157Q	B	F	IDU	B	42	194	4.04
S	S	S	P	P	None	E157Q	B	M	IDU	A	47	386	4.44
S	S	S	P	P	None	E157Q	B	F	HET	A	28	441	4.62
S	S	S	P	P	None	E157Q	B	M	UNK	UNK	37	UNK	UNK
S	S	S	P	P	None	E157Q	B	M	MSM	A	36	603	3.02
S	S	S	P	P	None	E157Q	B	M	UNK	UNK	42	UNK	UNK
S	S	S	P	P	None	T97A	B	M	HET	C	70	112	6.79
S	S	S	P	P	None	E157Q	B	M	MSM	A	50	982	4.74
S	S	S	P	P	None	E157Q	B	M	MSM	C	36	51	5.73
S	S	S	P	P	None	E157Q	B	M	IDU	C	40	191	4.72
S	S	S	P	P	None	E157Q	B	F	HET	C	44	184	4.92
S	S	S	P	P	None	E157Q	B	F	UNK	UNK	61	UNK	UNK
S	S	S	P	P	None	E157Q	B	M	UNK	UNK	58	UNK	UNK
S	S	S	P	P	None	E157Q	B	M	UNK	UNK	35	UNK	UNK

S	S	S	P	P	None	E157Q	B	M	UNK	UNK	40	UNK	UNK
S	S	S	P	P	None	E157Q	B	M	IDU	C	48	18	4.94
S	S	S	P	P	None	E157Q	B	M	HET	B	34	135	4.47
S	S	S	P	P	None	E157Q	B	M	HET	A	40	431	4.99

P- Potential low-level resistance
S- Susceptible

Figure S2. Prevalence of minor DRMs over time among men (a) and women (b)

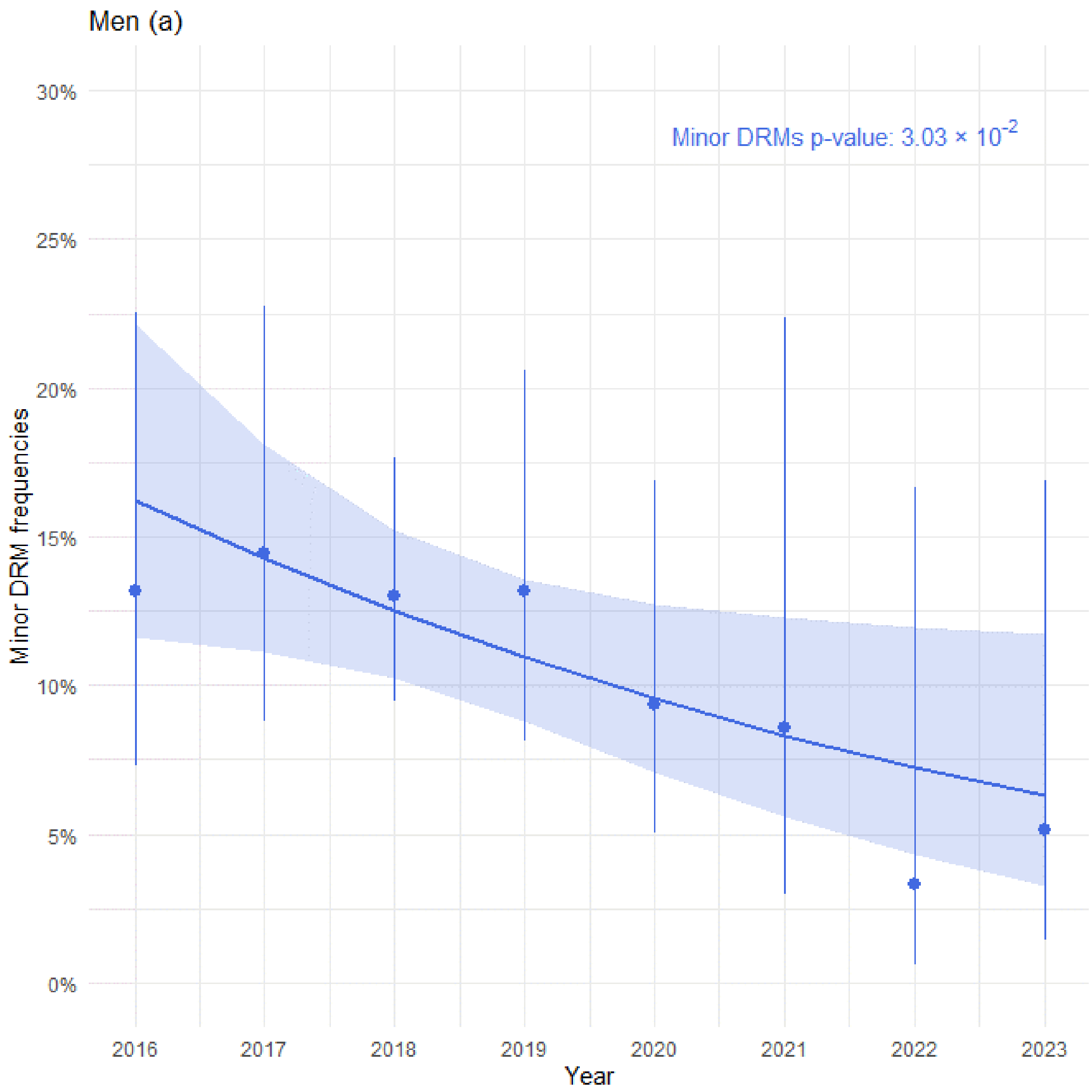

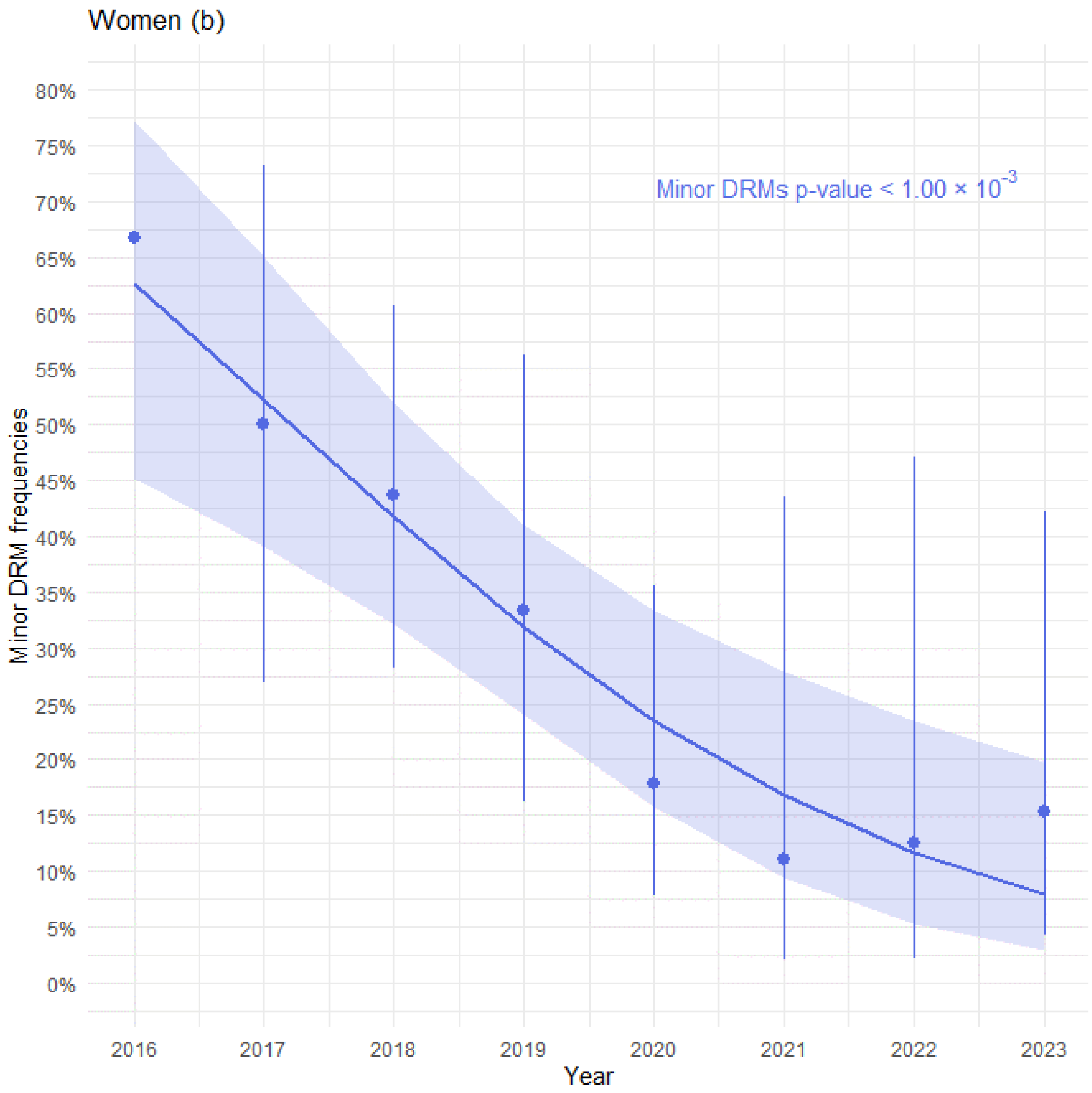

Women (b)
Minor DRMs p-value < 1.00 × 10-3
80%
75%
70%
65%
60%
55%
50%
45%
40%
35%
30%
25%
20%
15%
10%
5%
0%
Minor DRM frequencies
2016
2017
2018
2019
2020
2021
2022
2023
Year

Figure S3. Prevalence of minor DRMs over time among MSM (a), HET (b) and IDU (c)

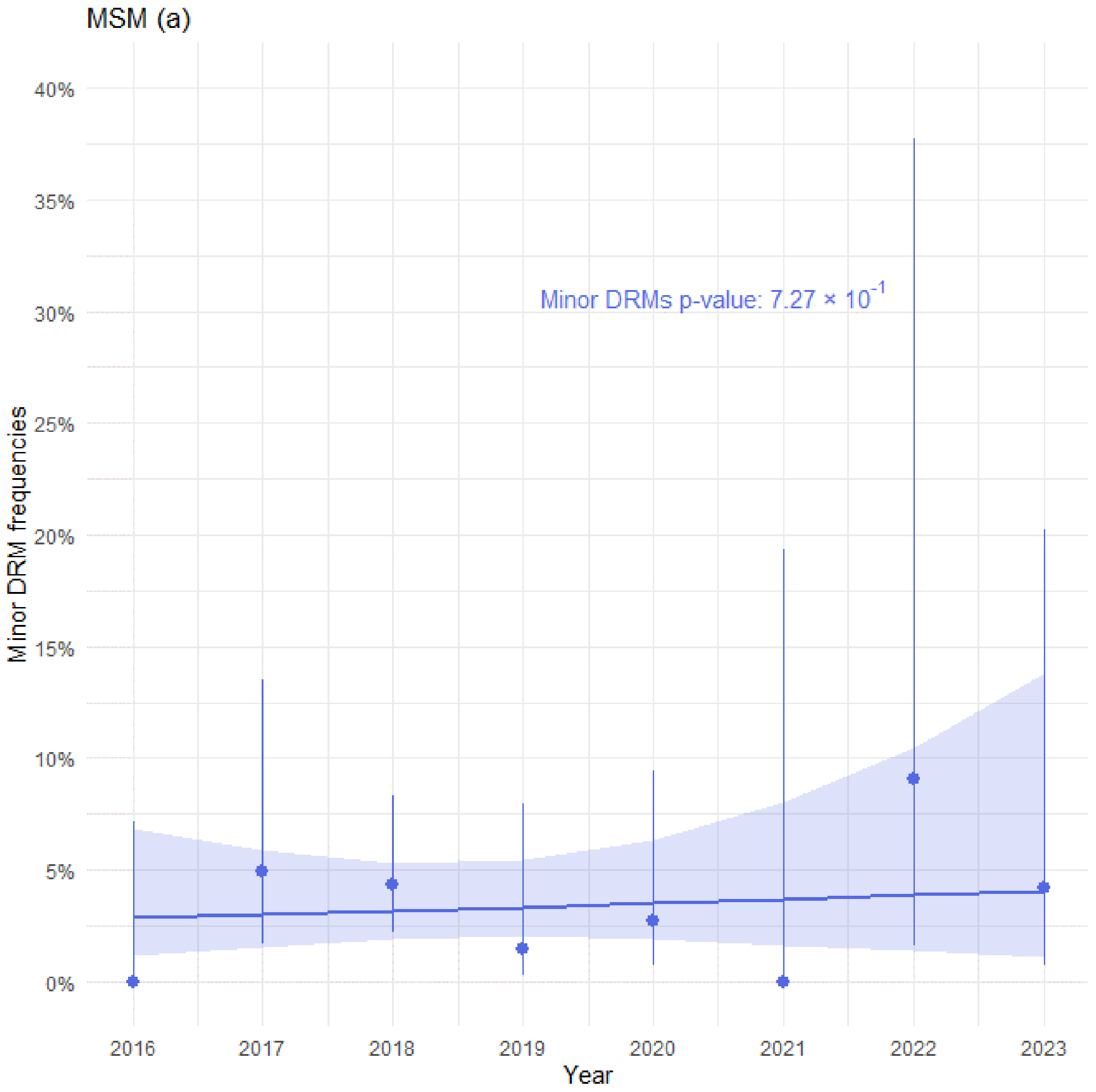

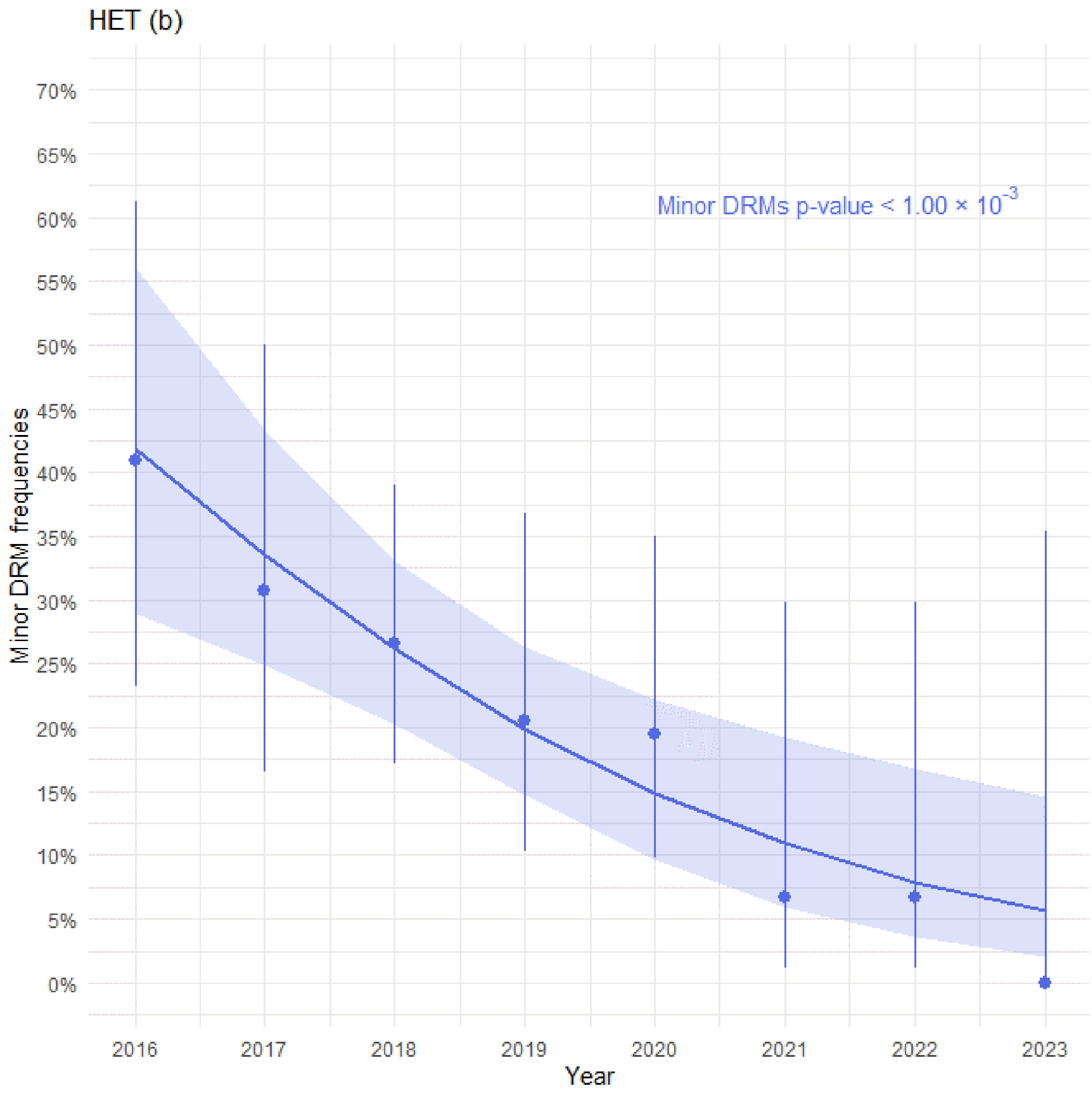
HET (b)
Minor DRMs p-value < 1.00 × 10^-3
Minor DRM frequencies
70%
65%
60%
55%
50%
45%
40%
35%
30%
25%
20%
15%
10%
5%
0%
2016
2017
2018
2019
2020
2021
2022
2023
Year

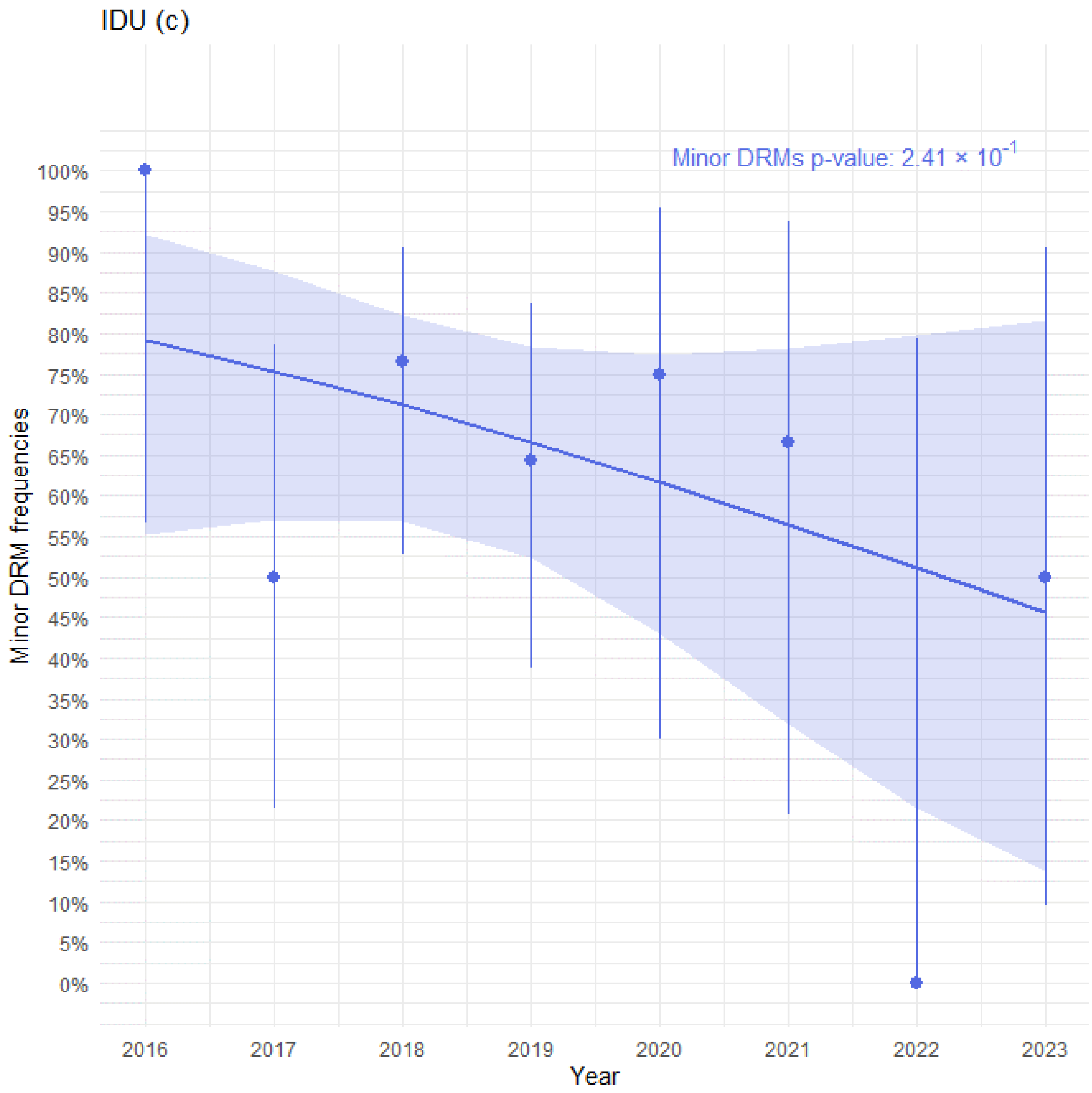

IDU (c)
Minor DRMs p-value: 2.41 × 10-1
100%
95%
90%
85%
80%
75%
70%
65%
60%
55%
50%
45%
40%
35%
30%
25%
20%
15%
10%
5%
0%
Minor DRM frequencies
2016
2017
2018
2019
2020
2021
2022
2023
Year
